# Supplementary figures and images for: Fludarabine–Cyclophosphamide-Based Conditioning with Antithymocyte Globulin Serotherapy Is Associated with Durable Engraftment and Manageable Infections in Children with Severe Aplastic Anemia
Source: J Clin Med. 2021 Sep 26;10(19):4416. doi: 10.3390/jcm10194416 (PMC8509585; doi:10.3390/jcm10194416)

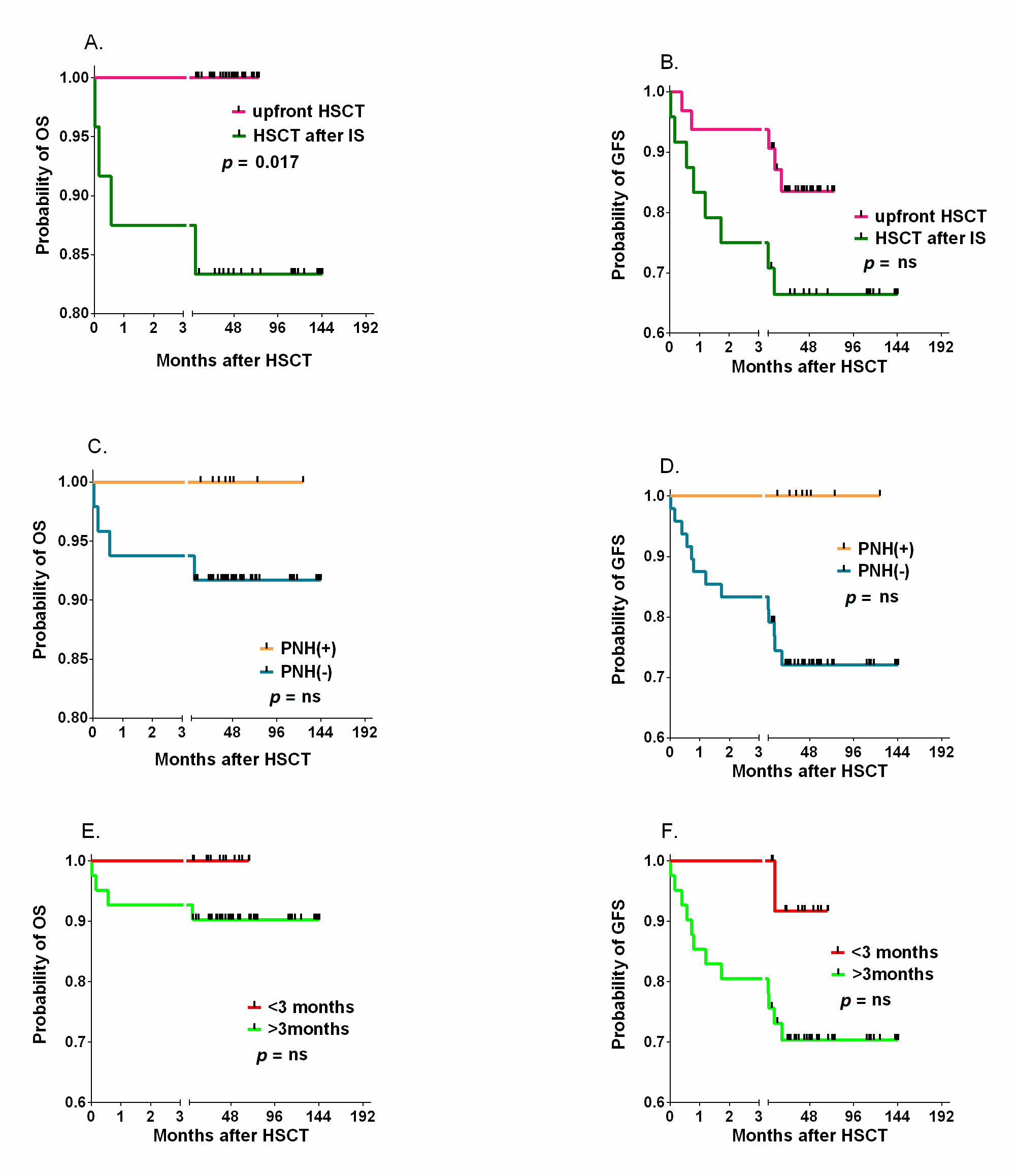

Supplement: Supplementary file 1 [file jcm-10-04416-s001.zip › NEW Suppl. Fig S1 26.09.tif]

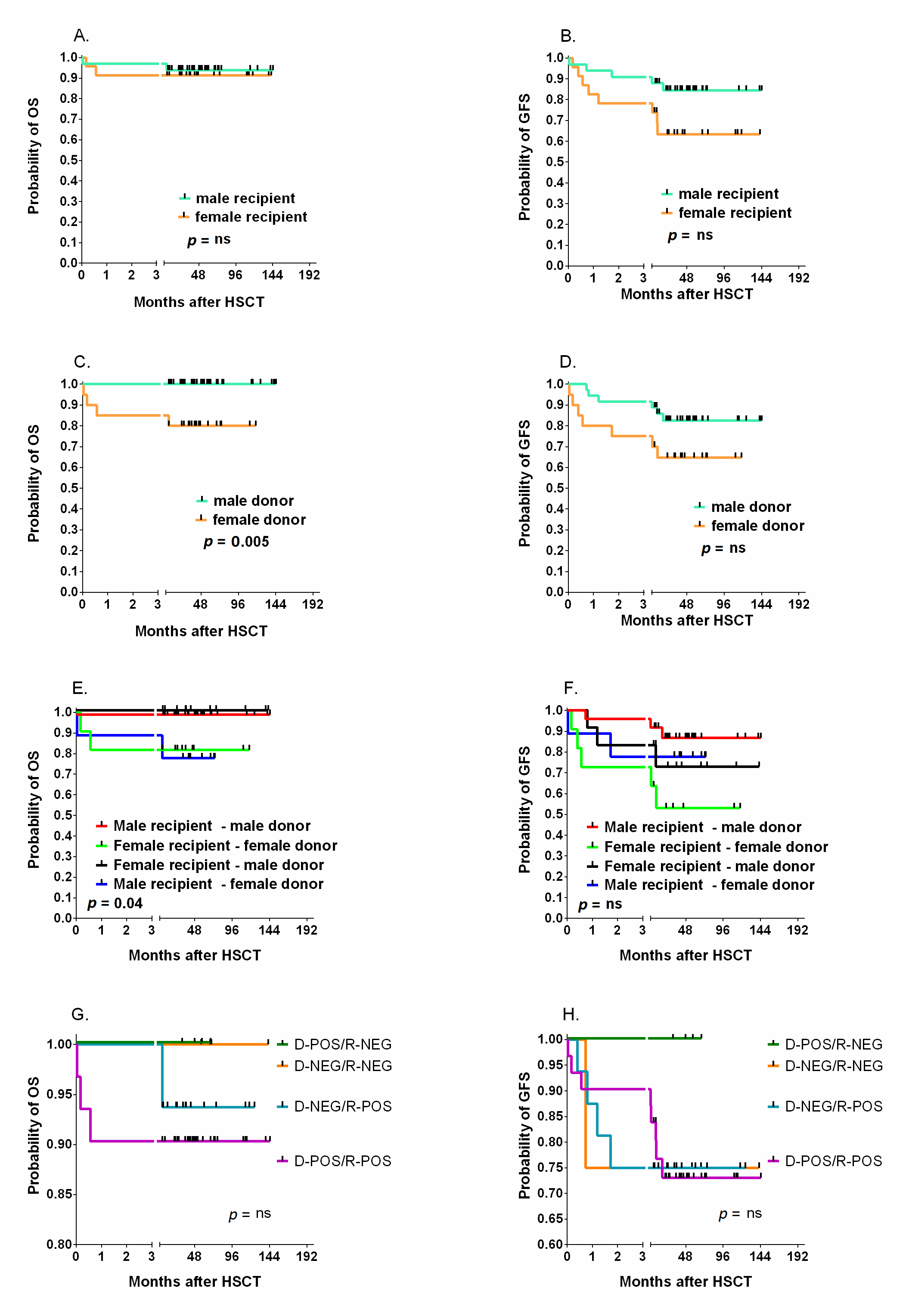

Supplement: Supplementary file 1 [file jcm-10-04416-s001.zip › NEW Suppl. Fig S2 26.09.tif]

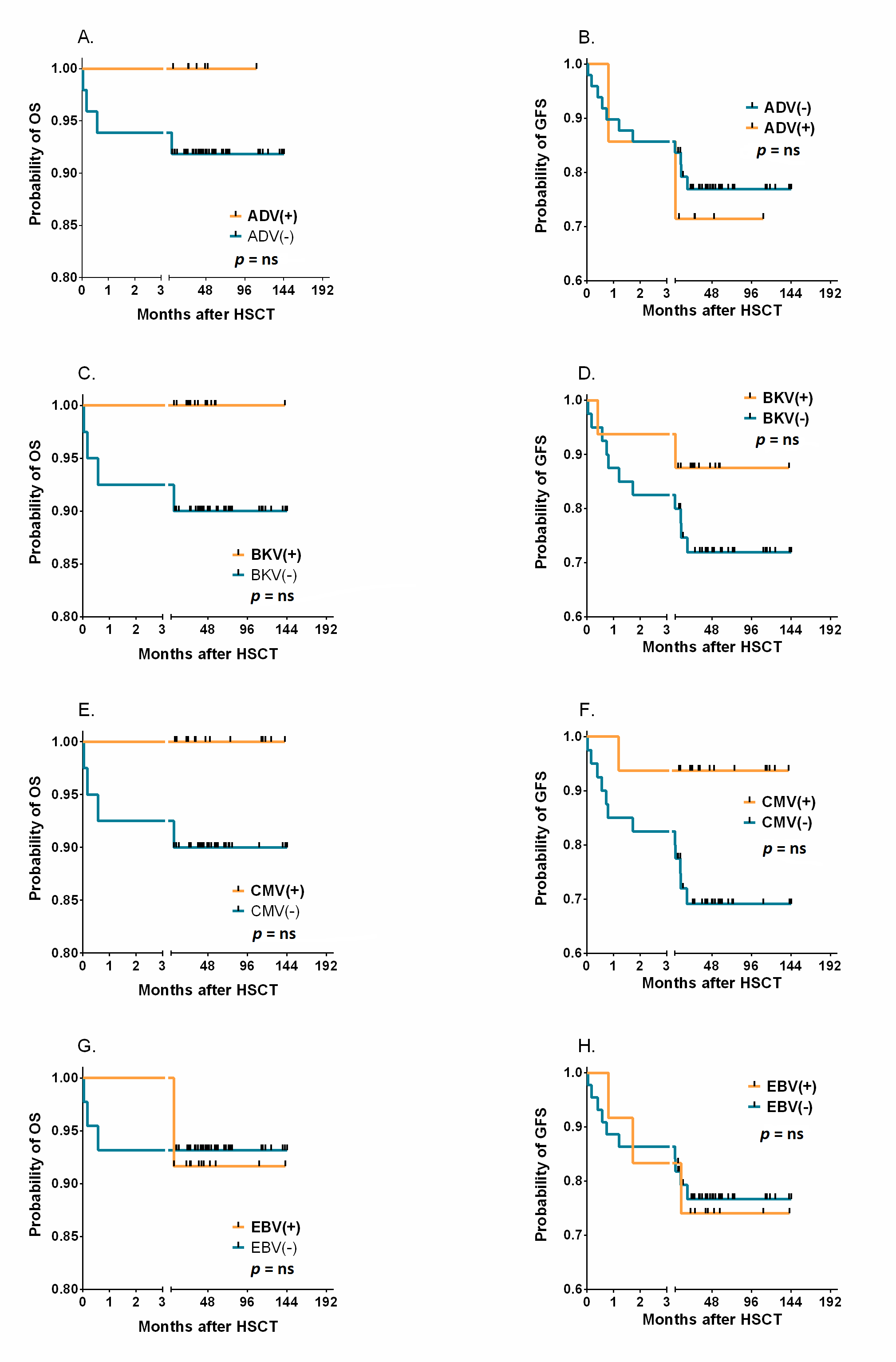

Supplement: Supplementary file 1 [file jcm-10-04416-s001.zip › Suppl. Fig S4 26.09.tif]

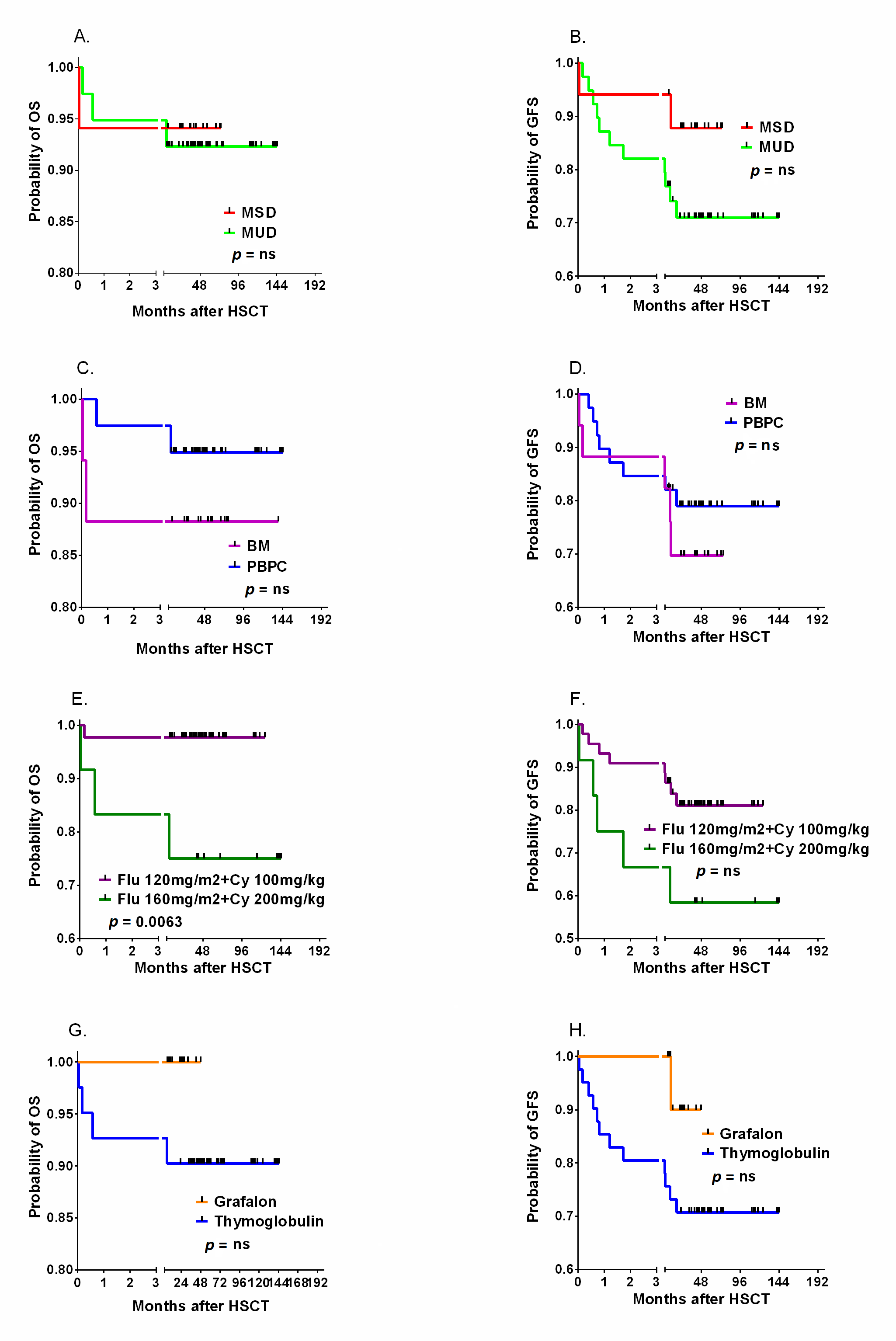

Supplement: Supplementary file 1 [file jcm-10-04416-s001.zip › Suppl.Fig S3 26.09.tif]
